# A cross-sectional survey unveiling the imperatives for continuing education and discipline development in pain medicine

**DOI:** 10.3389/fmed.2025.1541403

**Published:** 2025-04-02

**Authors:** Ren Jiang, Hong Li, Zhiyou Peng, Yanfeng Zhang, Xianhui Kang, Zhiying Feng

**Affiliations:** ^1^Department of Anesthesiology, Yinzhou No. 2 Hospital, Ningbo, China; ^2^Department of Pain Medicine, The First Affiliated Hospital, Zhejiang University School of Medicine, Hangzhou, China; ^3^Department of Anesthesiology, The First Affiliated Hospital, Zhejiang University School of Medicine, Hangzhou, China

**Keywords:** pain medicine, continuing medical education, advanced training, survey, disciplinary development

## Abstract

**Objective:**

This study aims to investigate persistent gaps in pain medicine education and unmet training needs, while exploring the significance of continuing education in driving disciplinary evolution.

**Methods:**

A questionnaire was distributed online in the form of an e-Questionnaire link to the directors of the Pain Medicine Departments of 417 hospitals (covered all hospitals) in Zhejiang Province in China. This questionnaire aimed to identify the problems and needs in continuing education for pain medicine. Subsequently, a questionnaire link was sent to 163 physicians nationwide who had undergone advanced training in the Pain Medicine Department to survey the existing problems and needs in advanced training.

**Results:**

The survey revealed uneven development of pain medicine, with secondary hospitals notably lagging in pain clinic establishment (51.3% vs. 69.9% in tertiary hospitals). The number of pain physicians is insufficient, and their overall academic qualifications need to be improved. Most directors (81.9%) have a strong willingness to enhance their professional capabilities, recommending advanced training. The number of advanced trainee has increased significantly, most physicians said that inpatient teaching accounts for about 3/4 of the advanced training duration. Case-based learning is the most popular between instructors (93.3%) and advanced trainees (82.2%). 46% of physicians reported having no opportunities for independent or semi-independent outpatient consultation, highlighting insufficient clinical practice opportunities. Additionally, Most physicians (93.3%) are satisfied with their instructors.

**Conclusion:**

The findings from this cross-sectional survey underscore the pressing need for a more robust and standardized continuing education framework in pain medicine in China.

## Introduction

Chronic pain has been recognized as an independent disease and included in the ICD-11 classification directory (1). Compared to other chronic diseases, chronic pain is characterized by a high incidence, low alleviation rate, and a greater disease burden (2). Data from the United Nations Statistics indicate that the incidence of chronic pain in adults ranges from 35.0 to 51.3%, and the Global Burden of Disease Study (2016 edition) reported that pain-related diseases are a major cause of global disability and disease burden (3). Chronic pain is a pervasive and costly problem; in the United States, the number of people affected even exceeds the combined total of diabetes, heart disease, and cancer, resulting in an annual economic loss of up to $635 billion. Despite such a high level of investment, 40 to 70% of patients still do not receive high-quality pain management (4). Non-specialist pain physicians often lack the theoretical knowledge and technical skills to effectively manage chronic pain, making it difficult to identify, correctly diagnose, and implement effective treatments (5, 6). Pain medicine specializes in diseases of pain, and pain physicians are the main force in the prevention and treatment of chronic pain diseases. The discipline of pain medicine has the responsibility to rapidly develop and alleviate patients’ pain, improve patients’ quality of life, and significantly reduce the public health burden of pain (7).

Pain medicine has developed rapidly, but to date it has been unable to meet society’s demand for pain assessment and treatment, leading to international calls for attention to pain medicine education (8). Medical education is the core of the development of a discipline and an effective way to enhance physicians’ theoretical knowledge, skills, and professional attitudes. The National Health and Family Planning Commission, in conjunction with the Ministry of Education and others, has proposed an orderly connection between undergraduate education, postgraduate education, and continuing education to achieve standardized and specialized physician training. Globally, most medical schools have not yet included pain medicine as a required course in their curriculum (9). Pain medicine residency training programs have not yet been established independently. In South Korea, anesthesiologists are the main practitioners of pain management, and their training as pain physicians is primarily through anesthesiology and pain medicine residency training (1 year), with a curriculum that emphasizes anesthesiology courses and weakens pain training (10). Continuing medical education (CME) is very common among doctors in North America, with about one-third of the U.S. anesthesiology training (3 years) dedicated to pain learning (11). After completing anesthesiology residency training, an additional year of pain medicine residency training is required to be certified as a pain physician. In China, the integration of pain medicine into medical education curricula remains limited; the standardized system for talent cultivation has not yet been established; the specialized training has not been implemented. These reasons have resulted in the uneven development of pain medicine.

Talent cultivation is key to maintaining the development of a discipline and addressing new challenges (12). To gain an in-depth understanding of the existing problems and needs in pain medicine continuing education, the Zhejiang Provincial Medical Association Pain Branch conducted a questionnaire survey among the directors of Pain Medicine Department in Zhejiang Province and physicians who had undergone advanced training in the Pain Medicine Department.

## Methods

### Questionnaire design and validation

The questionnaire used in this study was meticulously designed to ensure its relevance and validity. Prior to the full-scale survey, a pilot test was conducted with 30 physicians to refine the clarity and relevance of the questions. Feedback from the pilot test was used to make necessary adjustments to the questionnaire, ensuring that it effectively captured the required information. Additionally, the questionnaire underwent an expert review by 5 pain medicine specialists to ensure content validity. These experts provided valuable insights and recommendations, which were incorporated into the final version of the questionnaire. This rigorous design and validation process helped to ensure the reliability and accuracy of the data collected.

### Data collection

From January 2022 to June 2023 (Stage I), the Pain Medicine Branch of Zhejiang Province Medical Association organized an invitation for 417 hospitals (covered all hospitals) in Zhejiang Province to complete the ‘Zhejiang Province Pain Medicine Continuing Education Questionnaire’. The content of the questionnaire mainly aimed to investigate the existing problems and needs in continuing education for pain medicine. An e-Questionnaire link was sent to the directors of the Pain Medicine Departments.

From July 2023 to April 2024 (Stage II), physicians nationwide who had undergone advanced training in the Pain Medicine Department were invited to complete the ‘Advanced training Questionnaire of the Pain Medicine Department’. Relevant links were distributed online. The questionnaire included three aspects: general information of advanced trainees, relevant data of advanced training, and evaluations of instructors, in order to understand the existing problems and needs in pain medicine advanced training.

### Statistical analysis

Data processing and statistical analysis were conducted using the SPSS 25.0 software package. Prior to analysis, all quantitative data were subjected to tests for normality. The one-sample Kolmogorov–Smirnov test (One-Sample K-S test) was selected to assess the normality of the distribution of continuous variables. For quantitative data that conformed to a normal distribution, the mean ± standard deviation (Mean ± SD) was utilized as the descriptive statistic, and comparisons between groups were performed using the independent samples t-test. Quantitative data not conforming to a normal distribution were represented by the median (lower quartile, upper quartile) [Median (Q1, Q3)], and intergroup comparisons were made using the non-parametric Mann–Whitney U test. Qualitative data were expressed by frequency and percentage (number of cases, percentage, *n*, %), and group comparisons were conducted using the chi-square (χ^2^) test. In all statistical tests, a *p*-value less than 0.05 (*p* < 0.05) was considered to indicate statistical significance, thereby suggesting a significant difference between groups. The questionnaire demonstrated high internal consistency, with a Cronbach’s *α* coefficient of 0.948 for the trainee survey, indicating excellent reliability.

## Results

A total of 416 questionnaires of the ‘Zhejiang Province Pain Medicine Continuing Education Questionnaire’ were collected, with a valid recovery rate of 99.8%. For the ‘Advanced training Questionnaire of the Pain Medicine Department’, the participating physicians were from 156 hospitals across 21 provinces in China. A total of 163 questionnaires were collected, with a 100% valid recovery rate.

### General information of pain physicians in Zhejiang Province

In the 416 hospitals of Zhejiang Province, 44.7% (186 out of 416) were tertiary hospitals, and 55.3% (230 out of 416) were secondary hospitals. 59.6% (248 out of 416) had established pain clinics, with 69.9% (130 out of 186) of tertiary hospitals and 51.3% (118 out of 230) of secondary hospitals offering pain clinics. The proportion of secondary hospitals with established pain clinics was significantly lower than that of tertiary hospitals (χ^2^ = 14.759, *p* < 0.001). There were a total of 825 pain physicians, with 75.2% (620 out of 825) being male and 24.8% (205 out of 825) being female. 73.1% (603 out of 825) had undergraduate degrees, and 24% (198 out of 825) held master’s degrees or above, of which 94.0% (186 out of 198) were employed by tertiary hospitals and 6.0% (12 out of 198) by secondary hospitals, indicating a generally lower level of education among pain physicians in secondary hospitals. The percentage of those with senior professional titles was 60.2% (497 out of 825), with 38.8% (320 out of 825) working in tertiary hospitals and 21.4% (177 out of 825) in secondary hospitals, showing a lower incidence of senior professional titles among pain physicians in secondary hospitals (χ^2^ = 416.000, *p* < 0.001) (Table 1).

**Table 1:** General information of pain physicians in Zhejiang Province.

Basic information		Tertiary hospital	Secondary hospital	Total
Gender	Male	400 (48.5%)	220 (26.7%)	620 (75.2%)
	Female	147 (17.8%)	58 (7.0%)	205 (24.8%)
Age	<30	61 (7.4%)	19 (2.3%)	80 (9.7%)
	30 to 40	193 (23.4%)	76 (9.2%)	269 (32.6%)
	40 to 50	199 (24.1%)	132 (16.0%)	331 (40.1%)
	50 to 60	79 (9.6%)	45 (5.5%)	124 (15.1%)
	>60	15 (1.8%)	6 (0.7%)	21 (2.5%)
Degree	Doctorate	28 (3.4%)	0 (0%)	28 (3.4%)
	Master	158 (19.1%)	12 (1.5%)	170 (20.6%)
	Bachelor	353 (42.8%)	250 (30.3%)	603 (73.1%)
	Associate or below	8 (1.0%)	16 (1.9%)	24 (2.9%)
Professional Title	Senior	320 (38.8%)	177 (21.4%)	497 (60.2%)
	Intermediate	144 (17.5%)	70 (8.5%)	214 (26.0%)
	Junior	83 (10.1%)	31 (3.7%)	114 (13.8%)

### Data collected from Zhejiang Province pain medicine continuing education questionnaire

The survey results indicated that only 26.2% (65 out of 248) of the Pain Medicine Department directors were comparatively/very satisfied with the comprehensive diagnostic and treatment capabilities of their physicians, while 81.9% (203 out of 248) of the directors had a comparatively/very strong willingness to enhance their diagnostic and treatment abilities. The majority of the directors stated that the primary means of continuing education was through advanced training (60.9%), with the main purpose of advanced training being to improve diagnostic abilities (58.9%) and therapeutic operations (38.7%). For physicians with some experience, the most recommended period for in-depth study of specialized techniques was 3 to 6 months of advanced training (57.7%). If there were short-term training opportunities, most expressed the greatest interest in ultrasound-guided interventional treatments (38.7%) and radiofrequency treatment techniques (24.6%) (Table 2).

**Table 2:** Zhejiang Province pain medicine continuing education questionnaire.

Questions	Options	Tertiary hospitals	Secondary hospitals	Total
Are you satisfied with the professional capabilities	Very satisfied	4 (1.6)	2 (0.8)	6 (2.4)
	Satisfied	35 (14.1)	24 (9.7)	59 (23.8)
	Somewhat satisfied	31 (12.5)	29 (11.7)	60 (24.2)
	Not satisfied	59 (23.8)	64 (25.8)	123 (49.6)
Willingness to enhance professional capabilities	Very strong	40 (16.1)	34 (13.7)	74 (29.8)
	Strong	65 (26.2)	64 (25.8)	129 (52.0)
	Moderate	24 (9.7)	19 (7.7)	43 (17.4)
	None	0 (0.0)	2 (0.8)	2 (0.8)
Primary avenues for continuing education among young physicians.	Internship departmental	83 (33.5)	68 (27.4)	151 (60.9)
	Teaching by senior teachers	28 (11.3)	13 (5.2)	41 (16.5)
	Short-term training academic	12 (4.8)	19 (7.7)	31 (12.5)
	Conferences	6 (2.4)	19 (7.7)	25 (10.1)
The primary focus of pain physicians’ continuing education.	Diagnosis	74 (29.8)	72 (29.0)	146 (58.9)
	Therapeutic procedures	50 (20.2)	46 (18.5)	96 (38.7)
	Ward	3 (1.2)	1 (0.4)	4 (1.6)
	Scientific research	2 (0.8)	0 (0.0)	2 (0.8)
Specialized technical training is most recommended for in-depth study.	Training for 3 to 6 months	63 (25.4)	80 (32.3)	143 (57.7)
	Short-term specialized training	36 (14.5)	23 (9.3)	59 (23.8)
	Training for 1to 3 months	27 (10.9)	14 (5.6)	41 (16.5)
	Other	3 (1.2)	2 (0.8)	5 (2.0)
If there is short-term specialized technical training available, what direction is your department most interested in?	Ultrasound-guided intervention	39 (15.7)	57 (23.0)	96 (38.7)
	Radiofrequency therapy	40 (16.1)	21 (8.5)	61 (24.6)
	Treatment of soft tissue pain	17 (6.8)	21 (8.5)	38 (15.3)
	Spinal endoscopy techniques	13 (5.2)	9 (3.6)	22 (8.8)
	Cancer pain management	6 (2.4)	7 (2.8)	13 (5.2)
	Collagenase	9 (3.6)	0 (0.0)	9 (3.6)
	Other	5 (2.0)	4 (1.6)	9 (3.6)

### General information of advanced trainees

The results showed that from 2013 to 2015, the number of advanced trainees was 9 individuals (5.5%), while from 2016 to 2018, the number increased by 244% to 31 individuals (19%). Furthermore, from 2019 to 2021, there was a 210% increase to 96 individuals (58.9%) compared to the period from 2016 to 2018. Of these, 74.2% (121 out of 163) were male and 25.8% (42 out of 163) were female, with an average age of 41.3 ± 8.3 years. 76.1% (124 out of 163) held a bachelor’s degree, 20.8% (34 out of 163) held a master’s or doctoral degree, 45.4% (74 out of 163) had a senior professional title, and 45.4% (74 out of 163) had an intermediate professional title. 50.9% (83 out of 163) of the advanced trainees were from county-level medical institutions, 69.9% (114 out of 163) had been engaged in clinical pain diagnosis and treatment for more than 2 years, 49.7% (81 out of 163) of the physicians had a training period of 6 months, and 58.3% (95 out of 163) recommended a training duration of 6 months. 66.9% (109 out of 163) of the advanced trainees aimed to train in radiofrequency treatment, and 65.0% (106 out of 163) aimed to train in ultrasound-guided interventional treatment (Table 3).

**Table 3:** General information of advanced trainees.

Items	Options	Participants (*n*)	%
Advanced train time	Before 2012	27	16.6
	2013 to 2015	9	5.5
	2016 to 2018	31	19.0
	2019 to 2021	96	58.9
Gender	Male	121	74.2
	Female	42	25.8
Age	25 to 35	40	24.5
	36 to 45	82	50.3
	46 to 55	35	21.5
	>55	6	3.7
Highest degree	Doctorate	7	4.3
	Master	27	16.5
	Bachelor	124	76.1
	Associate	5	3.1
Professional title	Senior	74	45.4
	Intermediate	74	45.4
	Junior	15	9.2
Hospital affiliation	Provincial level	18	11.5
	Municipal level	62	39.7
	County level	76	48.7
Working years	≤ 2	49	30.1
	2 to 5	32	19.6
	5 to 10	37	22.7
	≥ 10	45	27.6
Duration of advanced train	1 to 2 months	12	7.4
	3 months	41	25.2
	6 months	81	49.7
	12 months	26	16.0
	other	3	1.8
Recommended duration of advanced train	1 to 2 months	2	1.2
	3 months	11	6.8
	6 months	95	58.3
	12 months	53	32.5
	other	2	1.2
Purpose of advanced train	Radiofrequency therapy	109	66.9%
	Ultrasound-guided intervention therapy	106	65.0%
	Transforaminal endoscopic spine surgery	58	57.1%
	Enhancing research capabilities	56	34.3%

### Relevant data of the advanced training of the Pain Medicine Department

The survey results indicated that 35.5% (53 out of 163) of physicians reported that outpatient teaching accounted for a quarter of their schedule, while 57.1% (93 out of 163) stated that inpatient teaching made up three-quarters. Furthermore, 45.4% (74 out of 163) of physicians mentioned that the teaching hospitals implemented tutor system. A significant majority, 81.0% (132 out of 163), reported that teaching hospitals organized 1 to 2 times per week of various forms of centralized learning. Additionally, 81.6% (133 out of 163) of physicians noted that instructors primarily used case-based teaching methods, and 82.2% (134 out of 163) expressed a preference for case-based teaching methods.

On average, 76.7% (125 out of 163) of physicians reported seeing fewer than 3 patients independently or semi-independently per day, with 46% (75 out of 163) stating that no outpatient opportunities were arranged. Only 30.7% (50 out of 163) reported seeing an average of 1 to 2 patients per day. Moreover, 72.4% (118 out of 163) of physicians had fewer than 2 opportunities per day to perform minimally invasive treatments, with 35% (57 out of 163) reporting no such opportunities. A mere 37.4% (61 out of 163) indicated an average of 1 case per day. Regarding research training, 31.3% (51 out of 163) of physicians stated that the advanced training hospitals did not arrange for scientific research training, and 41.7% (68 out of 163) reported that no medical humanities education was scheduled during their advanced training (Table 4).

**Table 4:** Relevant data of the advanced training of the Pain Medicine Department.

Items	Options	Participants (*n*)	%
Is tutor system implemented?	Yes	74	45.4
	No	89	54.6
Percentage of time spent on outpatient teaching	10%	45	27.6
	25%	53	32.5
	35%	28	17.2
	50%	20	12.3
	75%	17	10.4
Percentage of time spent on inpatient teaching.	10%	18	11.0
	25%	9	5.5
	35%	3	1.8
	50%	40	24.5
	75%	93	57.1
Opportunities for independent or semi-independent consultations.	None	75	46.0
	1–2 cases/day	50	30.7
	3–5 cases/day	19	11.7
	5–10 cases/day	7	4.3
	Over 10 cases/day	12	7.4
Opportunities for minimally invasive interventions under the guidance of teaching instructors.	None	57	35.0
	1 case per day	61	37.4
	2 to 5 case per day	40	24.5
	6 to 10 case per day	4	2.5
	≥11 case per day	1	0.6
instructors employ which teaching model?	Case-based learning	133	81.6
	Traditional PPT	95	58.3
	Multidisciplinary collaborative	56	34.4
	No fixed pattern	53	32.5
	Inquiry-based	51	31.3
Which teaching model do you prefer?	Case-based learning	134	82.2
	Multidisciplinary collaborative	79	48.5
	Inquiry-based	62	38.0
	Traditional PPT	41	25.2
Research training	None	51	31.3
	Once a week	30	18.4
	Twice a month	7	4.3
	Once a month	13	8.0
	Irregular	62	38.0
Medical humanities education	Yes	95	58.3
	No	68	41.7

### Evaluation of instructors by advanced trainees

A high level of satisfaction was reported by 93.3% (152 out of 163) of advanced trainees with the teaching capabilities of their instructors; similarly, 93.3% (152 out of 163) were very or relatively satisfied with the instructors’ professionalism. The enthusiasm of instructors was considered very or relatively high by 92.6% (151 out of 163). The classroom atmosphere was deemed very or relatively lively by 87.1% (142 out of 163), and 89% (145 out of 163) found the arrangement of teaching content to be very or relatively reasonable. Additionally, 83.4% (136 out of 163) believed that instructors were very or relatively adept at fostering clinical thinking skills. An overwhelming 96.3% (157 out of 163) felt that the advanced training played a very or relatively significant role in enhancing their professional capabilities (Table 5).

**Table 5:** Evaluation of instructors by advanced trainees.

Items	Options	Participants (*n*)	%
The teaching instructor’s instructional abilities are evaluated positively.	Extremely satisfied	94	57.7
	Very satisfied	58	35.6
	Moderately satisfied	8	4.9
	Not satisfied	2	1.2
	Not satisfied at all	1	0.6
The teaching instructor demonstrates a high level of dedication to teaching.	Extremely dedicated	104	63.8
	Very dedicated	48	29.5
	Moderately dedicated	10	6.1
	Not very dedicated	0	0
	Not dedicated at all	1	0.6
The teaching instructor delivers lectures with enthusiasm, capturing the attention of the audience.	Extremely enthusiastic	104	63.8
	Very enthusiastic	47	28.8
	Moderately enthusiastic	11	6.8
	Not very enthusiastic	1	0.6
	Not enthusiastic at all	0	0
The teaching instructor encourages interaction and maintains an engaging atmosphere during the lectures.	Extremely active	81	49.7
	Very active	61	37.4
	Moderately active	19	11.7
	Not very active	2	1.2
	Not active at all	0	0
The teaching content is logically arranged and presented in a clear and understandable manner.	Extremely reasonable	80	49.1
	Quite reasonable	65	39.9
	Moderately reasonable	15	9.2
	Not reasonable	3	1.8
	Not at all reasonable	0	0
The teaching instructor emphasizes cultivating clinical thinking.	Extremely proficient	75	46.0
	Quite proficient	61	37.4
	Moderately proficient	22	13.5
	Not proficient	5	3.1
	Not at all proficient	0	0
The teaching content is highlighted effectively and easy to summarize and grasp.	Very prominent	81	49.7
	Quite prominent	64	39.3
	Moderately prominent	15	9.2
	Not prominent	2	1.2
	Not at all prominent	1	0.6
How satisfied are you with the guidance and correction provided by the teaching instructor in editing medical documents?	Very satisfied	87	53.4
	Satisfied	61	37.4
	Neutral	2	1.2
	Dissatisfied	13	8.0
	Very dissatisfied	0	0
How satisfied are you with the guidance provided by the teaching instructor during clinical practice operations?	Very satisfied	91	55.8
	Satisfied	64	39.3
	Neutral	6	3.7
	Dissatisfied	0	0
	Very dissatisfied	2	1.2
How satisfied are you with the clinical thinking training and practical teaching provided by this teaching instructor?	Very satisfied	87	53.4
	Satisfied	67	41.1
	Neutral	7	4.3
	Dissatisfied	1	0.6
	Very dissatisfied	1	0.6
Would you like this teaching instructor to continue providing guidance?	Very strong	83	50.9
	Strong	66	40.5
	Moderate	11	6.8
	Weak	1	0.6
	Very weak	2	1.2
Does the content taught by the instructor align with your learning objectives and requirements?	Highly consistent	81	49.7
	Moderately consistent	69	42.3
	Somewhat consistent	10	6.1
	Not consistent	2	1.2
	Not at all consistent	1	0.6
Does continuing education	Very useful	102	62.6
contribute to improving professional capabilities?	Useful	55	33.7
	Generally useful	5	3.1
	Have no use	1	0.6

## Discussion

This study represents the first comprehensive investigation into the current state of continuing education in pain medicine within Zhejiang Province in China, covering virtually all hospitals in the province. The findings show that there is an uneven development of the pain discipline and a strong demand for pain continuing education. Notably, these results basically represent the current situation of pain medicine in the developed regions of China. International professional pain organizations have emphasized the importance of incorporating pain medicine into undergraduate medical curricula (13). In contrast, the current study indicates that in the process of promoting the integration of pain medicine into medical education in China, there is still considerable room for improvement. This underscores the urgent need to scale up pain management infrastructure and improve the education of pain physicians. In addition, the uneven development of the pain discipline is similar to tertiary hospitals, with only 51.3% (118 out of 230) establishing pain clinics, a result consistent with the 2022 survey on the construction of pain disciplines in China (14). An epidemiological investigation shows that chronic pain is prevalent in China, some volunteers indicated that they have not received professional pain treatment, which is largely related to the unevenness of pain medicine and the lack of confidence in pain treatment (15). The adverse impacts of pain on patients are not limited to pain itself and functional impairments, but also bring about obvious social impacts (16). As the main force of chronic pain, pain medicine must enhance their comprehensive clinical service capabilities. While strengthening top-level design for discipline development, it is also necessary to balance the construction of the discipline in grassroots hospitals and establish standards and specifications for discipline construction.

With a total population of 65.77 million in Zhejiang Province and only 825 pain physicians, of which 76.1% have a bachelor’s degree, it is evident that there is a shortage of pain physicians and that academic qualifications need to be improved in Zhejiang Province. There is a significant gap in the demand for continuing education in pain medicine. Talent cultivation is the top priority for the development of the pain discipline, and China is still in the exploratory stage of establishing a standardized and systematic talent training system (17). There is no standardized training for pain medicine residents, with only a 3–4 month rotation in Pain Medicine Departments arranged in the standardized training of anesthesiology. Similarly, there is no specialized physician training, and it is urgent to establish a standardized training system for pain medicine residents and specialized physicians at the national level. Secondary hospitals account for 37.7% (278 out of 825) of pain physicians, with only 1.5% having high academic qualifications and 21.4% holding senior professional titles. In grassroots hospitals, most physicians lack systematic training in pain medicine, making it difficult to fulfill the role of health guardians. At this stage, it is necessary to focus on the importance of continuing education for the development of the pain discipline.

**Figure 1:**
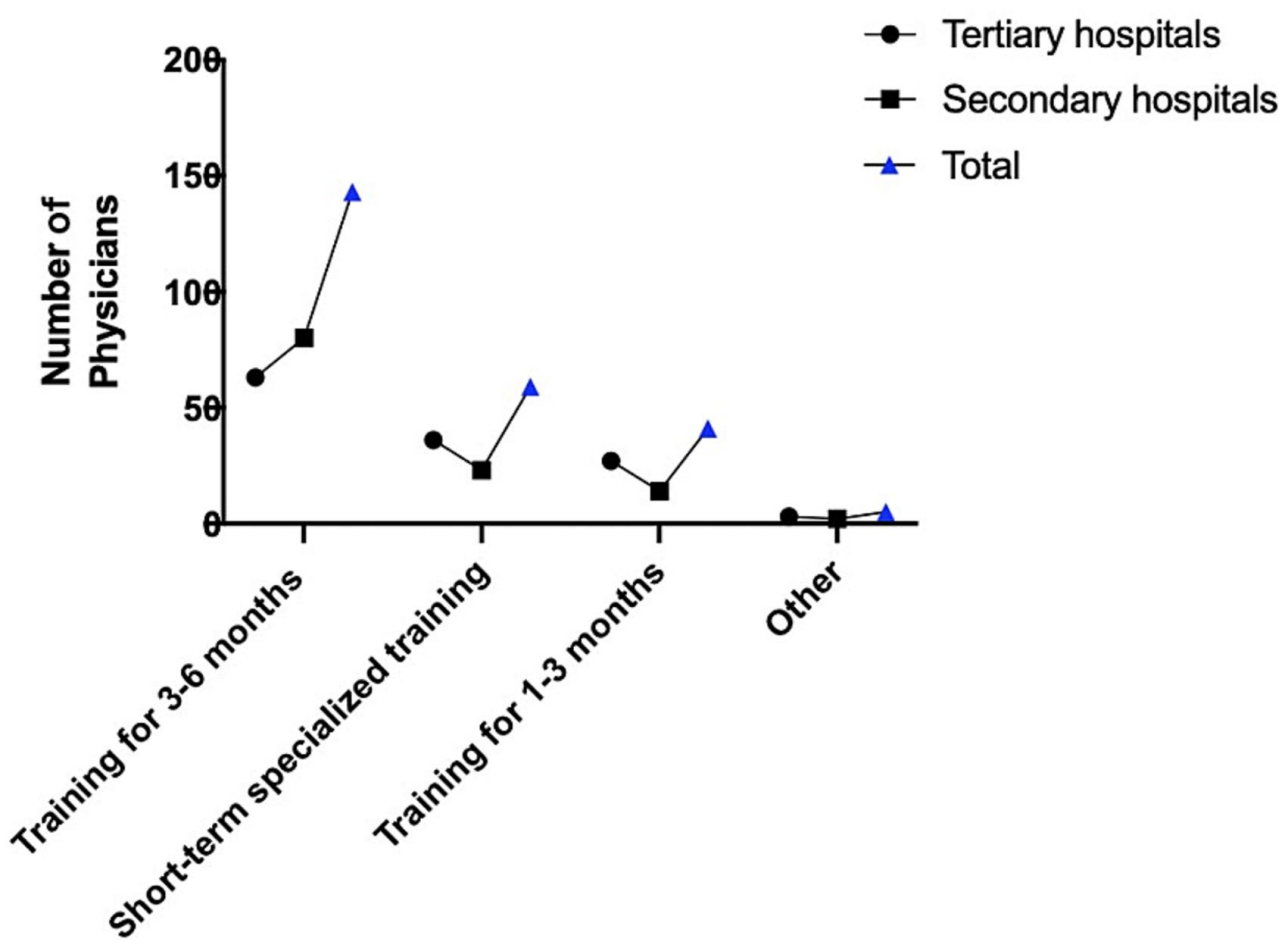
Recommendations for specialized technical training duration by directors of pain medicine departments in Zhejiang Province (*n* = 248).

The survey shows that most directors are not satisfied with the professional capabilities of their physicians, and they have a strong desire to improve, recommending of advanced training. It was illustrated in Figure 1 that the strong preference among department directors for 3 to 6 months of advanced training, with 57.7% recommending this duration. The preference for a 3 to 6 month training duration is based on key considerations that make this time frame optimal for enhancing physicians’ competencies. This duration provides comprehensive exposure to both theoretical knowledge and practical skills, which is crucial in pain medicine where minimally invasive techniques and advanced diagnostics are essential. It also aligns with the natural learning progression, starting with foundational knowledge and moving to complex clinical scenarios, ensuring a solid base before advancing. To further understand the problems and needs in advanced training, we conducted a questionnaire survey again. Physicians participating in this survey came from 156 hospitals nationwide, distributed across 21 provinces in China, indicating that the results can reflect the problems and needs in pain advanced training to a certain extent. The results show that the demand for pain advanced training has increased significantly in recent years. Teaching hospitals undertaking teaching tasks generally pay attention to the quality of advanced training, but there are also many shortcomings. Advanced trainees have a relatively high level of satisfaction with their instructors.

The number of advanced trainee has increased significantly, with the number from 2019 to 2021 increasing by 210% compared to 2016 to 2018. The increase in demand for advanced training is a trend: (1) There is a large demand for the diagnosis and treatment of chronic pain; (2) Alleviating pain is a basic human right, and comfortable medical care is a requirement of social progress; (3) Pain medicine lacks a standardized talent cultivation system; (4) Tertiary hospitals’ evaluation requirements include the construction of Pain Medicine Departments. Advanced trainees are mostly young men, have lower academic qualifications, come from county-level hospitals, choose provincial teaching hospitals for advanced training, and mostly train for 6 months, mainly learning ultrasound-guided intervention and radiofrequency treatment techniques. The results of this survey indicate that the pain discipline is gradually being recognized. On the other hand, it shows the role of teaching hospitals in promoting discipline development, which requires attention to be paid to teaching quality.

The survey results show that inpatient teaching accounts for about 3/4 of the advanced training time, nearly half of the teaching hospitals implement tutor system, and almost all teaching hospitals hold various forms of centralized learning, indicating that teaching hospitals generally pay attention to teaching quality. Outpatient teaching focuses on updating theoretical knowledge, while inpatient teaching focuses more on deepening technical skills. The training time required for different levels of ability cultivation varies (17). The allocation of teaching time needs to be individualized and stratified, taking into account the abilities and objectives of advanced trainees. Medical education is highly specialized and focuses on the inheritance of experience. In terms of teaching models, the tutor system is recommended. The tutor system creates a closer teacher-student relationship and is an effective system for training excellent physicians. During the advanced training stage, instructors can implement the concept of whole-process education, which is not only reflected in clinical diagnostic and treatment abilities but also includes psychological concern, daily care, and career planning guidance (18). It also helps to establish a long-term effective contact after advanced trainees return to their position, and promoting the construction of grassroots pain disciplines. However, there are also shortcomings, such as limited teacher resources and insufficient instructors-trainees matching. Centralized learning is an effective guarantee to improve the quality of teaching, which is conducive to teaching hospitals to formulate standard course catalogs and systematically explain various common and difficult diseases, and standardize the practice of pain treatment operations. Advanced trainees have diverse backgrounds, and it is recommended to implement stratified teaching while centralized learning to improve the quality and efficiency of advanced training teaching.

Instructors often use case-based learning (CBL), traditional PPT teaching (lecture-based learning, LBL), multidisciplinary team teaching (MDT) and other teaching methods, among which CBL is most commonly used (81.6%) and most popular among advanced trainees (82.2%). CBL takes advanced trainee as the main body, and guides instructors to cultivate trainees’ active learning and promote the integration of theory and practice (19). LBL (58.1%) takes instructors as the main body, and advanced trainees passively receive, and teaching quality highly depends on the instructors’ ability (20). Active learning (AL) strategies are more conducive to the retention and application of new knowledge (21). MDT (48.5%) is conducive to breaking the theoretical barriers between disciplines and establishing a multidimensional theoretical system. Pain medicine includes diagnosis, treatment, and comprehensive management, the complexity of pain, multidisciplinary intersection, and the explosive growth of knowledge, which requires physicians to have a wide range of knowledge, including anatomy, pharmacology, rehabilitation medicine, and interventional treatment (22). A single teaching method cannot meet teaching needs. It is recommended that teachers diversify teaching methods, advocate active learning for advanced trainees.

Most advanced trainees said that there were few clinical practice opportunities in the advanced training. The scarcity of clinical practice is a common issue, which may be related to the insufficient comprehensive ability of advanced trainees, heavy clinical work of instructors, and insufficient teaching ability. Pain medicine education should focus on cultivating skills, knowledge, behavior, and attitudes required for independent practice (23). Pain medicine is an operational discipline, and few practical opportunities are in obvious contradiction with training needs. If advanced trainees fail to proficiently master pain treatment techniques, it may bring serious consequences to patients after returning to their posts. Advanced training must pay attention to effective teaching (24). It is recommended that instructors arrange appropriate clinical practice opportunities according to the ability of advanced trainees, gradually let go but keep an eye on, and ensure medical safety while providing high-quality teaching (25). Teaching hospitals with more practice opportunities are more conducive to the training of pain talents, more likely to attract advanced trainees.

Outstanding physicians also need to have scientific research capabilities and strengthen medical ethics literacy. It is not enough for the training of medical staff to reach low standards; the teaching plan of educational hospitals should pursue excellence (26). Most advanced trainees have a bachelor’s degree, lack postgraduate training, and most teaching hospitals have not included scientific research in the advanced training content. Scientific research is the core cornerstone of discipline development, having scientific research capabilities is an inevitable requirement for physician promotion, and enhancing physicians’ scientific literacy can strengthen physicians’ continuous development capabilities. Chronic pain patients are often accompanied by anxiety and depression, which requires attention to humanistic care in pain diagnosis and treatment, emphasizing care and respect for patients (27).

The vast majority of advanced trainees express a high level of satisfaction with the comprehensive evaluation of their instructors, and the advanced training has proven to be immensely beneficial for enhancing their capabilities in pain management. The faculty bears the significant responsibility of nurturing physicians, and their teaching proficiency and enthusiasm are pivotal in determining the overall standard of young physicians’ training. Pain medicine, being an emerging field, implies that the construction of a faculty body is relatively insufficient. Most teaching hospitals do not allocate additional time for teaching, and instructors are often required to balance their clinical duties and research tasks, making it challenging to fully commit to their teaching responsibilities. It is recommended that teaching hospitals establish a teaching evaluation system, offering rewards for excellent instructors. The content of the advanced training should establish a systematic curriculum and standardize the treatment operation processes to ensure a structured and effective learning experience. Furthermore, it is crucial to integrate continuous feedback mechanisms and regular updates to the training materials. This approach not only fosters a conducive learning environment but also supports the instructors in their dual roles.

Grasping the challenges and demands within pain medicine’s continuing education realm is essential for the development of discipline. The study’s constraints should be noted: Firstly, the survey is limited in scope, focusing solely on the status of continuing education for pain medicine in Zhejiang Province, aiming to uncover the foundational academic and instructional requirements of China’s pain physicians. Variances in the collected data suggest a need for broader research that encompasses a national perspective in subsequent efforts. Secondly, the questionnaire targeting advanced physicians, distributed via WeChat groups and personal networks specific to the pain medicine community, may introduce a degree of bias due to its non-representative sampling method.

## Conclusion

Pain medicine still needs to accelerate development to meet society’s demand. This study emphasizes the importance of continuing education for the development of pain medicine. Teaching hospitals generally pay attention to the quality of advanced training, and there is still a need to improve in providing clinical practice opportunities and teaching models. Scientific research ability and medical humanities education should be included in the advanced training to cultivate elite talents. Strengthening the construction of the teacher team and allocating sufficient resources is an important guarantee for maintaining teaching quality and promoting discipline development.

## Data Availability

The original contributions presented in the study are included in the article/supplementary material, further inquiries can be directed to the corresponding authors.
